# Major comorbid diseases as predictors of infection in the first month after hip fracture surgery: a population-based cohort study in 92,239 patients

**DOI:** 10.1007/s41999-024-00989-w

**Published:** 2024-05-22

**Authors:** Nadia Roldsgaard Gadgaard, Claus Varnum, Rob Nelissen, Christina Vandenbroucke-Grauls, Henrik Toft Sørensen, Alma Becic Pedersen

**Affiliations:** 1 Department of Clinical Epidemiology, Aarhus University and Aarhus University Hospital, Olof Palmes Allé 43-45, 8200 Aarhus N, Denmark; 2 Department of Orthopedic Surgery, Lillebaelt Hospital, Vejle, Denmark; 3 Department of Regional Health Research, University of Southern Denmark, Odense, Denmark; 4 Department of Orthopedics, Leiden University Medical Center, Leiden, The Netherlands; 5 Department of Medical Microbiology and Infection Control, Amsterdam University Medical Center, Amsterdam, The Netherlands

**Keywords:** Comorbidity, Epidemiology, Geriatrics, Hip fracture, Infections, Population based

## Abstract

**Purpose:**

Comorbidity level is a predictor of infection in the first 30 days after hip fracture surgery. However, the roles of individual comorbid diseases as predictors of infection remain unclear. We investigated individual major comorbid diseases as predictors of infection after hip fracture surgery.

**Methods:**

We obtained Danish population-based medical registry data for patients undergoing hip fracture surgery (2004–2018). Information was obtained on 27 comorbidities, included in various comorbidity indices, 5 years before surgery. The primary outcome was any hospital-treated infection within 30 days after surgery. Cumulative incidence of infection was calculated by considering death as competing risk. We used logistic regression to compute mutually adjusted odds ratios with 95% confidence interval for infection.

**Results:**

Of 92,239 patients with hip fracture, 71% were women, and the median age was 83 years. The most prevalent comorbidities were hypertension (23%), heart arrhythmia (15%), and cerebrovascular disease (14%). The 30-day incidence of infection was 15% and 12% among the total cohort and among patients with no record of comorbidities, respectively. Infection incidence was highest among patients with renal disease (24%), depression/anxiety (23%), and chronic pulmonary disease (23%), and lowest among patients with metastatic solid tumor (15%). Adjusted odds ratios of infection ranged from 0.94 [0.80–1.10] for metastatic solid tumor to 1.77 [1.63–1.92] for renal disease.

**Conclusion:**

Most comorbid diseases were predictors of infection after surgery for hip fracture. Awareness of patients’ comorbidity profiles might help clinicians initiate preventive measures or inform patients of their expected risk.

**Supplementary Information:**

The online version contains supplementary material available at 10.1007/s41999-024-00989-w.

## Key summary points

**Aim:**

To investigate individual major comorbid diseases as predictors of infection after hip fracture surgery.

**Findings:**

Infection risk was higher among patients having any comorbid disease compared to patients without comorbid disease. The most important predictors of infection after hip fracture surgery were renal disease, depression/anxiety, and chronic pulmonary disease.

**Message:**

Comorbid diseases varied in importance as predictors of infection after hip fracture surgery.

**Supplementary Information:**

The online version contains supplementary material available at 10.1007/s41999-024-00989-w.

## Introduction

Hip fracture is a serious and frequent injury. The global annual incidence of hip fracture was 14 million in 2019 [1] and is predicted to continue to increase until 2050 with the aging of the world’s population [2].

The global average total cost of healthcare and social care 1 year after hip fracture has been estimated to be $43,669 per patient [3]. In addition, the 1-year mortality has been reported to be approximately 30% [4, 5]. Complications are frequent after hip fracture surgery and have been associated with diminished health status and quality of life, high healthcare and social costs, and elevated mortality [3, 4, 6]. Infections, including pneumonia and urinary tract infection, are among the most frequent complications and highly associated with mortality after hip fracture surgery [7, 8]. From 2005 to 2016, the infection risk increased among patients with hip fractures, whereas a similar trend was not observed in the general population of the same age and sex [9]. This finding is of major concern, because the 30-day mortality among patients with hip fractures with any infection is more than twice that of infection-free patients with hip fractures [10]. Thus, accurate identification of patients with hip fractures at high infection risk is of increasing importance.

Comorbidity level, as measured by the Charlson comorbidity index (CCI) [11], is associated with elevated infection risk after surgery for hip fracture [12]. However, the CCI does not include potentially important predictors of infection, such as depression [13] or thyroid disease [14]. Furthermore, the CCI was developed for predicting mortality in a population of patients with breast cancer [11]. Therefore, the assigned weights used to calculate the CCI summary score may overestimate or underestimate the importance of the individual comorbid diseases in the context of infection in patients with hip fracture.

Relying on the comorbidity indices may mask the potential important effects of individual comorbid diseases, thus hindering stratified preventive interventions. However, relative and absolute measures of infection after hip fracture surgery based on individual comorbid diseases are rare in the current literature, although both measures are highly clinically relevant for evaluating effects on healthcare systems and patients. Several studies in patients with hip fracture have reported an association between selected comorbid diseases and the risk of specific infections [15–19], but with conflicting results [16–18]. Those studies have been hindered by methodological problems including small sample sizes [15–19], potential selection bias [15–19], and loss to follow-up [19]. Furthermore, few comorbid diseases or comorbidity measures have been evaluated [15–19].

Therefore, we investigated a wide selection of prevalent major comorbid diseases and their roles as predictors of infection within 30 days after hip fracture, using data from Danish health registries.

## Method

### Setting and data sources

This study was set in Denmark, which has approximately 5.9 million residents [20]. The tax supported Danish healthcare is universally accessible and includes general practitioners and hospitals. We obtained data from the following nationwide medical registries: 1) the Danish Multidisciplinary Hip Fracture Registry (DMHFR), which has recorded information on patient characteristics, surgery, quality indicators, and prognostic factors for all patients with hip fractures ≥ 65 years of age since 2004 [21, 22]; 2) the Danish Civil Registration System, which has recorded information on vital and civil status, and residence for all Danish residents since 1968, using a unique personal identifier assigned to all Danish residents enabling linkage between all Danish medical registries on individual level [23]; 3) the Danish National Patients Registry [24], which has recorded information on in-hospital treatments and surgical procedures performed, as well as 1 primary and as many as 19 secondary discharge diagnoses, since 1977, and information on outpatient clinic discharges and emergency department visits since 1995. The discharge diagnoses have been coded according to the International Classification of Diseases 10th Edition (ICD-10) since 1994.

### Study cohort

The study cohort was identified from the DMHFR and included 92,239 patients treated surgically for a first unilateral hip fracture between January 2004 and October 2018.

## Comorbid diseases

Comorbidity indices including the CCI [11], the Elixhauser Comorbidity Index [25], the Rx-risk Comorbidity Index [26], and the Nordic Multimorbidity Index [27] guided the selection and definition of chronic comorbid diseases, for example diabetes, and other comorbid conditions, for example anemia, as potential predictors. This was done to improve the comparability of our results to that of other studies using these indices. Information on comorbid diseases was obtained for each patient from the ICD-10 diagnosis codes recorded in the Danish National Patient Registry in the 5 years before the date of hip fracture surgery. Both primary and secondary diagnoses from in-hospital and outpatient clinic visits were used. Diagnosis codes recorded at emergency department visits were excluded, because of potentially low validity, given that non-specific symptom-based diagnoses are the most frequent diagnoses in Danish emergency department visits [28]. As comorbid diseases accumulate in patients over time, the prevalence increases with an increasing length of the lookback period. However, the length of the lookback period has been shown to have minimal effects on the ability of the comorbidity indices to predict infections among patients with hip fractures [29]. A lookback period of 5 years was chosen to ensure applicability in clinical practice, aiming to identify most patients’ comorbidity diagnoses, under the assumption that past information on diagnoses would become harder to collect in clinic with prolonged lookback period. To ensure statistical precision and clinical relevance, we included only comorbid diseases with a prevalence > 1%; consequently, comorbid diseases, such as HIV/AIDS (prevalence 0.02%) and hemiplegia (prevalence 0.2%), were not included. In total, 27 comorbid diseases were evaluated. For two diseases, a distinction was made by severity: patients with diabetes were categorized as either having uncomplicated or complicated diabetes, and patients with solid tumors were categorized by metastasis status, as having either no or any metastatic solid tumors. Further details, including diagnosis codes and descriptions of the diagnoses for each of the 27 comorbid diseases, are provided in Online Resource 1.

### Infection

The primary outcome was defined as any hospital-treated infection recorded in the Danish National Patient Registry. These infections included common and serious viral and bacterial infections, such as pneumonia, urinary tract infection, skin infection, surgical-site infection, or gastrointestinal infection, as well as rarer infections, parasitic or mycotic infections. The primary outcome was identified as primary and secondary discharge diagnoses of any type of infection during in-hospital and outpatient clinic visits. Infection diagnosis codes recorded during emergency department visits were also included, on the basis of the assumption that infection is an acute disease easily diagnosed in acute settings, according to characteristic symptoms (e.g., fever and cough) and readily accessible paraclinical tests (e.g., chest X-ray, or urine and blood samples), and can be treated in an emergency department setting [30]. The primary outcome of any hospital-treated infection is important from a patient perspective since any infection after hip fracture surgery is a serious complication associated with poorer prognosis [10]. Secondary outcomes of pneumonia and urinary tract infection were also identified because these infections are prevalent and are among the most common causes of death in patients with hip fracture and thus are of particularly high clinical relevance [7, 8]. Lists of diagnosis codes used for the definition of outcomes in this study are available in the Online Resource 1.

### Variables

Information on surgery date was collected from the DMHFR. From the Danish Civil Registration system, patient age and sex were collected at the surgery date, whereas dates of death or emigration were collected within the study period. Age was categorized into three groups: 65–74, 75–84, and ≥ 85 years.

### Statistical analysis

The number and prevalence of baseline characteristics were reported for the total cohort.

For both primary and secondary infection outcomes, patients were followed from baseline, defined as the date of surgery, until the date of infection, migration, death, or 30 days after surgery, whichever came first.

Numbers of incident infection cases and the cumulative incidence of infection, considering death a competing risk, were calculated for the total cohort, stratified by each selected comorbid disease, and for patients with no record of comorbid diseases [31].

Using logistic regression, we estimated crude odds ratios (ORs) for infection according to each of the selected comorbid diseases, comparing patients with each selected comorbid disease (i.e., exposed) to patients without the selected comorbid disease (i.e., unexposed) in 27 bivariate models. The ORs at baseline can be interpreted as the odds of infection within the first 30 days after surgery among exposed patients compared with unexposed patients (e.g., patients with vs. without a record of renal disease) [32].

In addition, a multivariate model including all 27 selected comorbid diseases, sex, and age as a continuous variable, was computed with logistic regression. From the multivariate model, mutual adjusted ORs as a measure of relative risks were estimated. These adjusted OR can be interpreted as the direct effect of an individual comorbid disease, adjusted for confounding and/or mediation through age, sex, and the other 26 comorbid diseases included in the model [33].

To evaluate possible effect modification by associated factors and allow for stratified evaluation of infection risk, we performed stratified analyses estimating the cumulative incidence and adjusted ORs by age and sex for the primary outcome.

Estimates were presented with 95% confidence intervals (CIs). Analysis was performed using R software (version 2022.02.1).

This article follows the Strengthening the Reporting of Observational Studies in Epidemiology (STROBE) and the Reporting of studies Conducted with Observational Routinely collected Data (RECORD) guidelines for cohort studies.

## Results

Among the total cohort (*n* = 92,239), the median age was 83 years, 71% of patients were women, and 36% had no record of comorbidity within the past 5 years. The most prevalent comorbid diseases were hypertension (23%), heart arrhythmia (15%), and cerebrovascular disease (14%), followed by chronic pulmonary disease, any solid tumors, and fluid/electrolyte disorders, with a prevalence of 10–11% (Table 1).

**Table 1:** Characteristics of the total cohort

Variable	*N* (%)
Total cohort	92,639 (100%)
Sex	
Female	65,565 (71.1%)
Male	26,674 (28.9%)
Age	
65–74 years	18,105 (19.6%)
75–84 years	35,618 (38.6%)
≥ 85 years	38,516 (41.8%)
Median age (interquartile range)	83 (77, 89)
Surgery year	
2004–2008	31,675 (34.3%)
2009–2013	31,702 (34.4%)
2014–2017	28,862 (31.3%)
Fracture type	
Fracture of head and neck of femur	48,922 (53.0%)
Pertrochanteric fracture of femur	36,771 (39.9%)
Subtrochanteric fracture of femur	6,546 (7.1%)
Surgery type	
Arthroplasty, hemi and total	28,998 (31.4%)
Osteosynthesis	63,241 (68.6%)
Comorbidity level	
Mild, CCI score 0	45,649 (49.5%)
Moderate, CCI score 1–2	33,614 (36.4%)
Severe, CCI score ≥ 3	12,976 (14.1%)
Median CCI score (interquartile range)	1.00 (0.00, 2.00)
Comorbid diseases	
No comorbid disease	33,341 (36.2%)
Cardiovascular	
Cerebrovascular disease	12,796 (13.9%)
Heart arrhythmia	13,967 (15.1%)
Heart failure	6,850 (7.4%)
Hypertension	21,235 (23.0%)
Hypotension	1427 (1.6%)
Myocardial infarction	3193 (3.5%)
Peripheral vascular disease	5476 (5.9%)
Valvular heart disease	3877 (4.2%)
Hepatic/gastrointestinal	
Liver disease	1045 (1.1%)
Peptic ulcer	3276 (3.6%)
Malignant	
Any solid tumor	9297 (10.1%)
Hematologic cancer	1080 (1.2%)
Metastatic solid tumor	1209 (1.3%)
Metabolic	
Diabetes, complicated	3621 (3.9%)
Diabetes, uncomplicated	3927 (4.3%)
Hypercholesterolemia	4420 (4.8%)
Thyroid disease	3492 (3.8%)
Musculoskeletal	
Rheumatic disease	3410 (3.7%)
Neurological/psychological	
Alcohol use disorder	2245 (2.4%)
Dementia	8074 (8.8%)
Depression/anxiety	4151 (4.5%)
Neurological disorder	4371 (4.7%)
Pulmonary	
Chronic pulmonary disease	9647 (10.5%)
Pulmonary circulation disorder	1388 (1.5%)
Renal/hematological:	
Anemia	8364 (9.1%)
Fluid and electrolyte disorder	9700 (10.5%)
Renal disease	3159 (3.4%)

*CCI* Charlson comorbidity index

### Any infection

A total of 14,199 patients were treated in hospital for any infection within the first 30 days after surgery, corresponding to a cumulative incidence of 15%. For patients with no record of any of the 27 comorbid diseases, the cumulative incidence of infection was 12%.

**Fig. 1:**
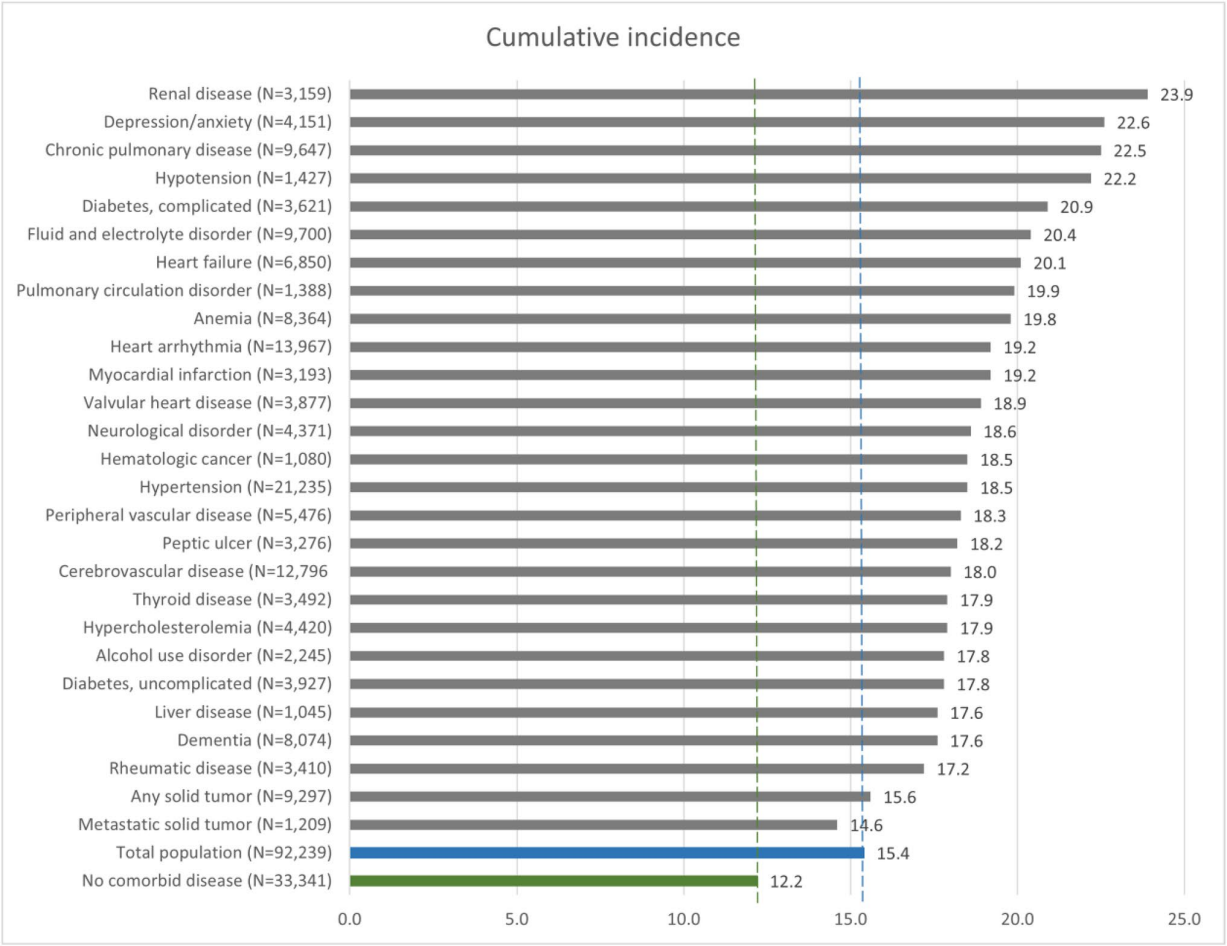
Bar chart plotting 30-day cumulative incidence of any infection for the total cohort, and with stratification by no comorbid disease or selected comorbidities

The cumulative incidence of infection was highest among patients with renal disease (24%), depression/anxiety (23%), and chronic pulmonary disease (23%). The lowest cumulative incidences were found among patients with metastatic solid tumors (15%), any solid tumors (16%), and rheumatic disease (17%) (Fig. 1).

**Fig. 2:**
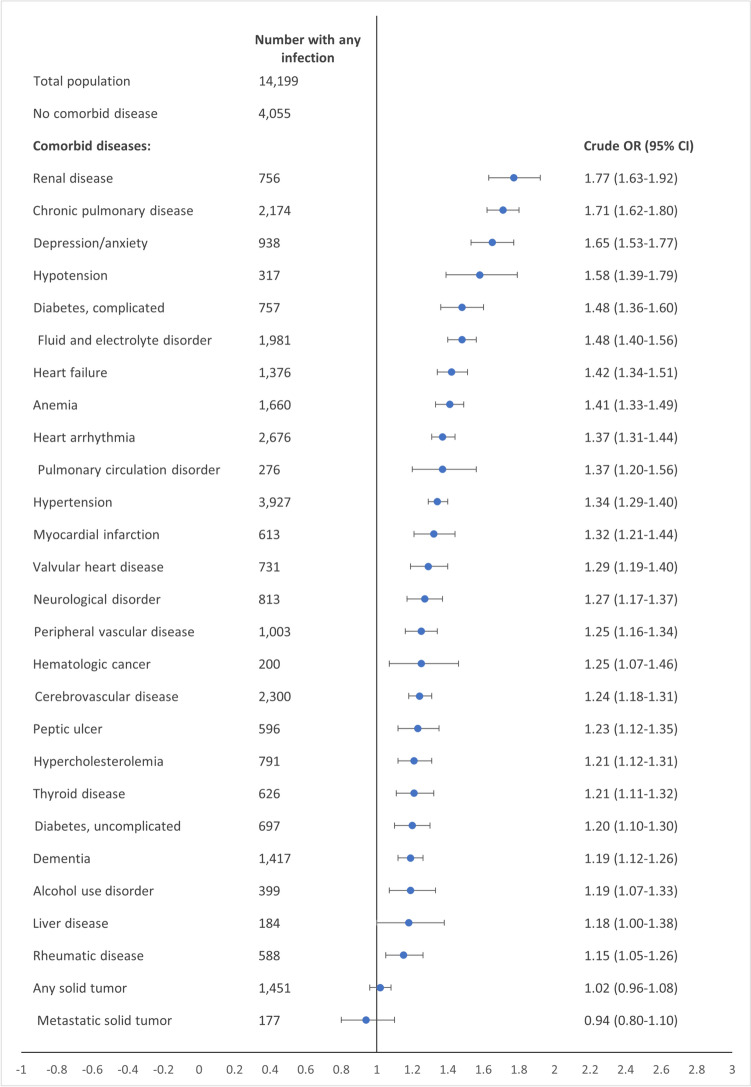
Forest plot of crude odds ratios for any infection, comparing patients with vs. without each selected comorbidity, at 30-day follow-up. *OR* odds ratio, *CI* confidence interval

The crude OR for infection varied between 0.94 (95% CI 0.80–1.10) for solid metastatic tumors and 1.77 (95% CI 1.63–1.92) for renal disease. The second, third, and fourth highest crude ORs were 1.71 (95% CI 1.62–1.80) for chronic pulmonary disease, 1.65 (95% CI 1.53–1.77) for depression/anxiety, and 1.58 (95% CI 1.39–1.79) for hypotension (Fig. 2).

**Fig. 3:**
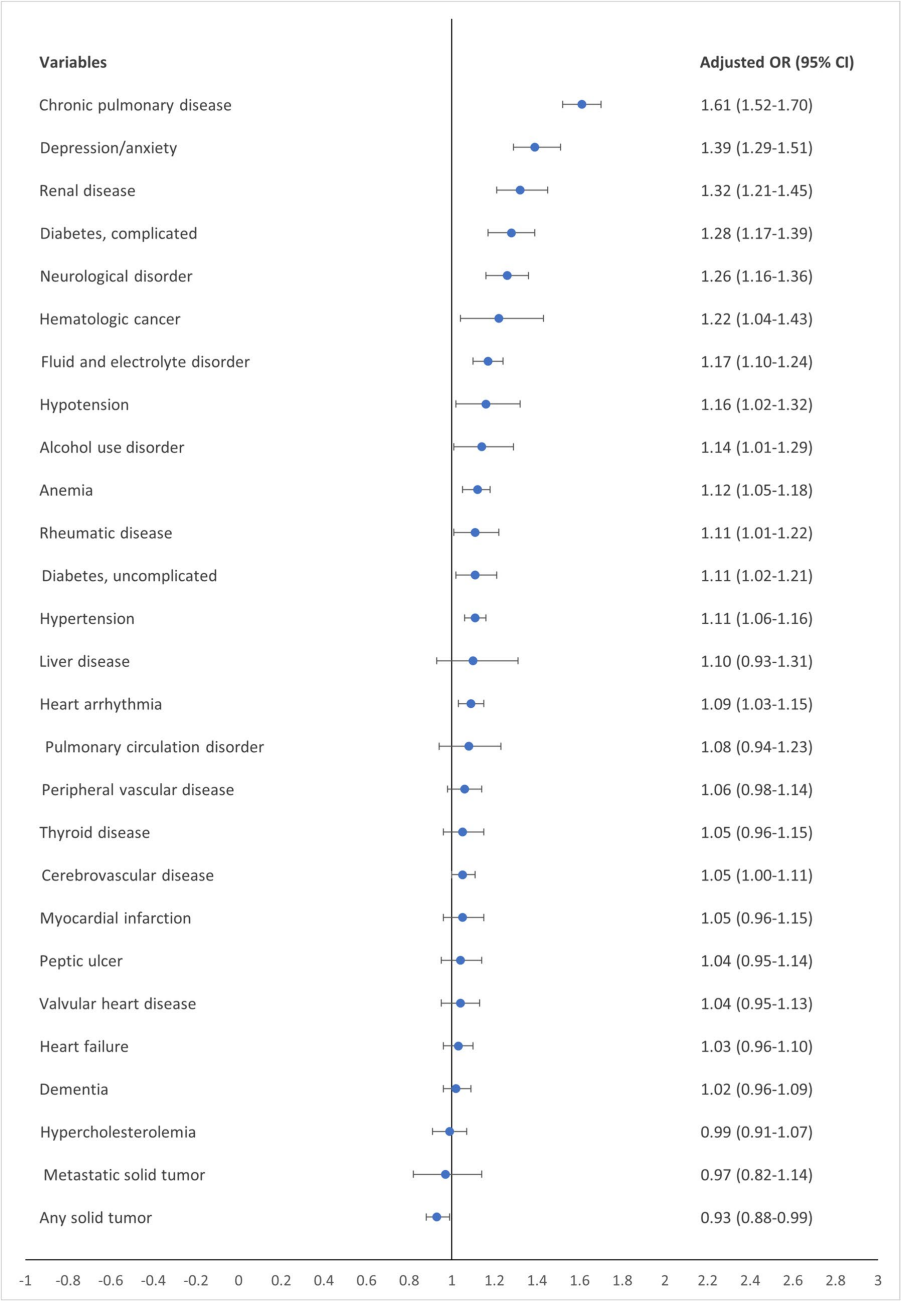
Forest plot of adjusted odds ratios for any infection at 30-day follow-up, mutually adjusted for age, sex, and comorbid diseases. *OR* odds ratio, *CI* confidence interval

The adjusted OR varied between 0.93 (95% CI 0.88–0.99) for any solid tumors and 1.61 (95% CI 1.52–1.70) for chronic pulmonary disease. The second, third, and fourth highest adjusted ORs were 1.39 (95% CI 1.29–1.51) for depression/anxiety, 1.32 (95% CI 1.21–1.45) for renal disease, and 1.28 (95% CI 1.17–1.39) for complicated diabetes (Fig. 3).

### Pneumonia

Within 30 days after surgery, 5784 patients had an incident hospital record of pneumonia. The cumulative incidence of pneumonia was 6% for the total cohort and 4% for patients with no comorbid disease. The highest cumulative incidence of pneumonia was observed among patients with chronic pulmonary disease (12%), renal disease (11%), and hypotension (10%).

The adjusted ORs varied between 0.83 (95% CI 0.62–1.09) for liver disease and 2.22 (95% CI 2.07–2.39) for chronic pulmonary disease (Online Resource 2).

### Urinary tract infection

In the first month postoperatively, 6614 patients had an incident hospital-treated urinary tract infection. The cumulative incidence of urinary tract infection was 7% for the overall cohort and 6% among patients with no comorbid disease. The cumulative incidence was highest among patients with depression/anxiety (11%) and hypotension (10%). The adjusted ORs varied between 0.81 (95% CI 0.62–1.04) for metastatic solid tumors and 1.41 (95% CI 1.26–1.56) for depression/anxiety (Online Resource 2).

### Stratified analysis

The cumulative incidence of infection for the total cohort and for patients with no comorbid disease increased with age, whereas cumulative incidence by selected comorbid disease increased or varied slightly across age groups, except for dementia, for which the cumulative incidence decreased with increasing age. In a comparison of the oldest patients (≥ 85 years) to the youngest patients (65–74 years), most adjusted ORs decreased with increasing age (Online Resource 2).

The cumulative incidence overall, among patients with no comorbid disease, and by selected comorbid diseases, was higher among male than female patients. In female patients, most adjusted ORs were similar to or higher than those in male patients (Online Resource 2).

## Discussion

Most of the selected chronic comorbid diseases were predictors of infection in the first postoperative 30 days among patients with hip fracture. The highest absolute risks of any infection were observed among patients with chronic pulmonary disease, renal disease, and depression/anxiety.

### Comparison to existing research

The prevalence of some of the investigated diseases was comparable to those previously reported (e.g., peripheral vascular disease of 5.9% vs. 4.6–8.9% [34–36]), while others differed noticeably (e.g., diabetes 8.2% vs. 12.5–24% or chronic pulmonary disease 10.5% vs. 12–23% [34–36]). These differences in prevalence are likely explained by differences between our study population and the populations of previous studies, including differences in age (aged ≥ 65 years vs. aged < 90 years [34] or aged ≥ 55 years [35]), gender (men and women vs. women only [35]), and nationality (Denmark vs. United States [34, 36] or Norway [35]). Additional explanations for the differences in prevalence include the applied methods and definitions used for the detection of comorbid diseases, e.g., data source (registry data vs. viewing medical records [34]), diagnosis codes (ICD-10 vs. ICD-9 [34, 36]), and lookback periods (5 years vs. 1–7 years [35] or undisclosed [34, 36]).

Few studies have evaluated the associations of one to five selected comorbid diseases and specific infections (e.g., pneumonia or urinary tract infection) among patients with hip fractures [15–19]. Our findings on pneumonia and urinary tract infection after hip fracture are in agreement with findings from some [15, 16, 19] but not all [15, 17, 18] previous studies. For example, the presence of diabetes as a predictor of pneumonia is supported by some previous studies [16] but not others [17, 18]. Discrepancies in the results between our study and previous studies are likely to be explained by differences in methodology, including differences in study type (e.g., non-intervention vs. intervention studies [18]), follow-up time (e.g., 30-day follow-up vs. in-hospital follow-up [17]), and measurement of outcomes (e.g., recorded diagnosis codes vs. requirement for antibiotic treatment [17, 18] or positive paraclinical tests suggesting infection [18]). In addition, differences in the sample size (92,239 vs. 86–806 patients [15–19]), selection of the study population (e.g., nationwide vs. single center [17, 18] or use of more exclusion criteria [18]), data collection, data availability, case mix (patients ≥ 65 vs. ≥ 70 [17] years of age), and healthcare setting might have introduced selection and information biases, or weakened the generalizability among study populations.

### Interpretation and perspectives

The associations we found may be explained through both causative mechanisms and factors such as age, sex, and lifestyle factors, and through association with other comorbidities [37]. For example, chronic pulmonary disease is a well-known risk factor for pneumonia and therefore, as expected, was a strong predictor of pneumonia in our study [38, 39]. We also found depression/anxiety to be an important predictor. Depression has been reported as a risk factor for infection; however, depression, particularly later in life, might be strongly associated with age-associated comorbid diseases [40, 41]. However, depression/anxiety remained an important predictor after adjustment for comorbid disease, age, and sex. Other factors, including smoking status, reduced immune status, and frailty might explain an increased vulnerability to infection among those with depression/anxiety [42–44].

The individual comorbid diseases could be considered markers of frailty or an underlying burden of disease [45, 46]. This could partially explain the associations observed for comorbid diseases that are not well-established risk factors for infection, such as hypotension, or comorbid diseases that are more transitory and potentially not present at time of surgery, e.g., fluid or electrolyte disorder or anemia. Frailty, which is prevalent in the older population, is generally described as a state of diminished reserve and physical stamina, as well as increased vulnerability to adverse health related outcomes [45]. Frailty is not synonymous with comorbidity or disability, although all three conditions are closely related [45]. We were not able to accurately measure frailty for several reasons. First, although tools for evaluating frailty exist and are increasingly implemented, particularly in geriatric medicine, consensus is lacking on one single well-defined and well-recognized measure of frailty [47, 48]. Second, in the available registries, frailty tests have not been documented. Third, the available tools for calculating frailty scores that would be applicable to our data rely largely on comorbidity status and other proxies [47]. Fourth, patients may present with serious comorbidity without being frail [45].

Unexpectedly, patients with comorbid diseases, such as metastatic solid tumors or dementia, had the lowest cumulative incidence of any infection. Metastatic solid tumors were associated to reduced infection risk in male patients, and dementia was associated to reduced risk in patients ≥ 85 years of age. However, these estimates appear to have been biased by the competing risk of death [49]. Factors such as delay in clinical recognition of infection or missed diagnoses, perhaps especially among those with dementia, metastatic tumors, or advanced age, could have contributed to an increased risk of mortality. Moreover, patients presenting with infections can be admitted to a hospital, treated in the community setting, or even given end of life care—a choice likely to depend on the patient’ comorbidities. Thus, our results are likely to have been influenced by the decisions regarding level of care.

In this study, we investigated comorbid diseases diagnosed or treated in the hospital setting. However, some milder comorbid diseases may be exclusively diagnosed and treated in general practice, and certain comorbid diseases are known to be underreported, e.g., dementia [50]. This could have contributed to the high cumulative incidence of infections among those we identified as having no comorbid disease.

Our results on the incidence and ORs for a wide range of chronic comorbid diseases may be relevant in clinical decision-making, considering patient-specific information, for decreasing infection rates in patients with hip fractures. Thus, clinicians might consider quality of care in patients with comorbidities, as previous studies reported an inverse association between increasing comorbidity level and fulfillment of quality-of-care indicators relevant for infection prevention, such as post-surgical mobilization [51, 52]. Interventions consisting of patient tailored programs including education and involvement of family caregivers or implementation of early transitional care programs to continue the multidisciplinary effort in the home-setting potentially could benefit patients at high infection risk [53, 54]. As such, the results of this study may help clinicians plan infection-preventive preoperative and postoperative care, initiate surveillance for early detection of infections, or inform patients of their expected infection risk.

### Methodological considerations

This study has limitations. First, our study cohort was based on data from the DMHFR [22]. Although the completeness and coverage of the DMHFR is unknown, reporting to the register is mandatory and partly automated, and orthopedic departments had economic incentives to report the surgeries performed in the entirety of the study period. Thus, the capture rate is believed to be high, and the selection bias is likely to be minimal.

Second, bias from misclassification of any variables cannot be excluded. The event of hip fracture surgery, as recorded in the DMHFR, has a positive predictive values (PPV) of 100% [22].

Although there is an economic incentive for the hospitals to report diagnoses to the DNPR, the validity of most comorbid diseases included in our study was evaluated previously using various population samples. The reported PPVs ranged between 80 and 100%, indicating a low level of over-reporting [24, 55–57]. It is unknown whether the PPVs of the remaining comorbid diseases in the DNPR are at the same level.

A study in patients with cancer has reported PPVs of 98% for any infection and 93% for pneumonia in the Danish National Patient Registry [58]. Infection diagnoses from emergency department contacts have been reported to have a PPV of 78% [30].

We did not have access to information from general practice consultation visits, because data on diagnoses given in general practice are not recorded in any registry. Therefore, this study was restricted to comorbid diseases and infections serious enough to require hospital contact. Consequently, our estimated prevalences of hospital-diagnosed comorbid diseases are lower than the prevalence would have been if we had been able to include the milder cases from general practice and undiagnosed cases. Under the assumption that hospital-diagnosed comorbid diseases are more severe than those solely diagnosed in general practice, these hospital-diagnosed comorbid diseases might be stronger predictors of severe infection and thus infection treated in hospital. However, the opposite may apply for certain comorbid diseases, e.g., severe dementia, where the clinician might opt for infection treatment in the community setting.

Third, the results of our study are best generalized to populations with similar case mix and from countries with similar healthcare setting and hip fracture treatment as that of our study population.

## Conclusion

Incident infection was present in 15% of patients in the first 30 days after hip fracture surgery. Moreover, 71% of patients with hip fractures with infection had one or more comorbid diseases. Our results indicated that most comorbid diseases are important predictors of a wide range of infections in patients within the first 30 days after surgery for hip fracture.

## Supplementary Information

Below is the link to the electronic supplementary material.

Supplementary file1 (PDF 210 KB)

Supplementary file2 (PDF 235 KB)

## Data Availability

To protect the privacy of patients, it is by Danish law prohibited to make individual level data publicly available.
