# Supplementary material for: Major comorbid diseases as predictors of infection in the first month after hip fracture surgery: a population-based cohort study in 92,239 patients
Source: Eur Geriatr Med. 2024 May 22;15(4):1069–80. doi: 10.1007/s41999-024-00989-w (PMC11377556; doi:10.1007/s41999-024-00989-w)
Supplement: Supplementary file 1 — Supplementary file1 (PDF 210 KB) [file 41999_2024_989_MOESM1_ESM.pdf]

Online Supplementary Material 1

Journal

European Geriatric Medicine

Article title

Major comorbid diseases as predictors of infection in the first month after hip fracture surgery: a population-based cohort study in 92,239 patients

Authors

Nadia Roldsgaard Gadgaard[1], Claus Varnum[2,3], Rob Nelissen[4], Christina Vandenbroucke-Grauls[1,5], Henrik Toft Sørensen[1], and Alma B. Pedersen[1]

[1] Department of Clinical Epidemiology, Aarhus University Hospital and Aarhus University, Denmark

[2] Department of Orthopedic Surgery, Lillebaelt Hospital – Vejle, Denmark

[3] Department of Regional Health Research, University of Southern Denmark, Denmark

[4] Department of Orthopedics, Leiden University Medical Center, The Netherlands

[5] Department of Medical Microbiology and Infection Control, Amsterdam University Medical Center, Amsterdam, The Netherlands

Corresponding Authors

Nadia Roldsgaard Gadgaard, MD, PhD student

Department of Clinical Epidemiology

Aarhus University and Aarhus University Hospital

Olof Palmes Allé 43-45

8200 Aarhus N, Denmark

E-mail: nrg@clin.au.dk

Appendix Table A – International Classification of Disease 10th Edition (ICD-10) codes used for defining comorbid diseases

COMORBID DISEASES	EXAMPLES AND ELABORATION ON COMORBID DISEASES	ICD-10 CODES
CARDIOVASCULAR:		
Cerebrovascular disease	Ischemia and haemorrhagic stroke; Transient ischemic attack; Etc.	I60-I69; G45-G46
Heart arrhythmia	Atrioventricular block; Paroxysmal tachycardia; Atrial fibrilation and flutter; Etc.	I44.1 - I44.3; I45.6; I45.9; I47-I49; R00.0; R00.1; R00.8; T82.1; Z45.0; Z95.0
Heart failure	Hypertensive heart disease; Ischemic cardiomyopathy; Myocarditis; Chronic ishemic heart disease; Heart failure; Etc.	I11.0; I13.0; I13.2; I09.9; I25.5; I42.0; I42.5 - I43; I50; P29.0
Hypertension	Essential hypertension; Hypertensive heart disease or kidney disease; Secondaye hypertension; Etc.	I10-I13; I15
Hypotension	Orthostatic hypotension; Etc.	I95; G90.9A
Myocardial infarction	Acute myocardial infarction; Complications to acute myocardial infarction; Etc.	I21-I23
Peripheral vascular disease	Atherosclerosis; Arotic or perophereal anoeurysm and dissection; Arterial embolism and thromobosis; Claudicatio; Etc.	I70-I74; I77
Valvular heart disease	Mitral and aortic valve disease; Etc.	A52.0; I05-I08; I09.1; I09.8; I34-I39; Q23.0-Q23.3; Z95.2-Z95.4
HEPATIC/GASTROINTESTINAL:		
Liver disease	Hepatitis; Esophegael varices; Alcoholic liver disease; Toxic liver diesase; Hepatic failure; Liver cirrhosis; Etc.	B15.0; B16.0; B16.2; B18; B19.0; I85; K70.0-K70.4; K70.9; K71-K74; K76.0; K76.6
Peptic ulcer	Ulcer of esophagus; Gastric, duodenal or gastrojejunal ulcer; Etc.	K22.1; K25-K28
MALIGNANT:		
Any solid tumor	Malignant neoplasm inlcuding brain cancer, lung cancer, breast cancer, melonoma; Etc.	C00-C75
Hematologic cancer	Lymphoma; Malignant myeloma; Leukemia; Etc.	C81-C85; C88; C90-C96
Metastatic solid tumor	Malignant neoplasm with mestastasis; Etc.	C76-C80
METABOLIC:		
Diabetes, complicated	Type I and II diabetes with kidney, ophthalmic, circulatory complication; Etc.	E10.2-E10.8; E11.2-E11.8
Diabetes, uncomplicated	Type I and II diabetes without complications	E10.0, E10.1; E10.9, E11.0; E11.1; E11.9
Hypercholesterolemia	Hypercholesterolemia; Etc.	E78.0

Thyroid disease	Iodine-deficiency; Non-toxiz goiter; Thyrotoxicosis; Hypothyroidism; Etc.	E00-05; E06.2; E89.0
MUSCULOSKELETAL:		
Rheumatic disease	Sarcoidosis; Rheumatoid arthritis; Vasculitis; Systemic lupus erythematosus; Systemic sclerosis; Ankylosing spondylitis; Etc.	D86; L94.0; L94.1; L94.3; M05-M06; M08-M09; M12.0; M12.3; M30-M36; M45; M46.1; M46.8; M46.9
NEUROLOGICAL/PSYCHOLOGICAL:		
Alcohol use disorder	Mental and behavioural disorders due to use of alcohol; Niacin deficiency; Alcoholic cardiomyopathy; Alcoholic liver disease; Etc.	F10; E52; G62.1; I42.6; K29.2; K70.0; K70.3; K70.9; T51; Z50.2; Z71.4; Z72.1
Dementia	Alzheimers dementia; Vascular dementia; Dementia in relation to neurological disorder; Etc.	F00-F03; F05.1; G30
Depression/anxiety	Bipolar disorder; Depression; Phobias; Generalized anxeity; Etc.	F20.4; F31.3-F31.5; F32-F34.1; F40-F41; F41.2; F43.2; F45.2
Neurological disorder	Huntington's disease; Parkinson's disease; Multiple sclerosis; Epilepsy; Encephalopathy; Etc.	G10-G13; G20-G22; G25.4; G25.5; G31.2; G31.8; G31.9; G32; G35-G37; G40-G41; G93.1; G93.4; R47.0; R56
PULMONARY:		
Chronic pulmonary disease	Bronchitis; Emphysema; Chronic obstructive pulmonary disease; Asthma; Pneumoconiosis; Interstitial lung disease; Etc.	J40-J47; J60-J67; J68.4; J70.1; J70.3; J84.1; J92.0; J96.1; J98.2; J98.3
Pulmonary circulation disorder	Pulmonary embolism; Pulmonary hypertension; Etc.	I26; I27; I28.0; I28.8; I28.9
RENAL/HAEMATOLOGICAL:		
Anemia	Deficiency anemia; Blood loss anemia; Aplastic anemia; Etc.	D50-59, D60-64
Fluid and electrolyte disorders	Volume depletion; Disorders of fluid, electrolyte and acid-base balance; Etc.	E22.2, E86-E87
Renal disease	Hypertensive chronic kidney disease; Glumerular disease; Renal tubulo-interstitial diseases; Kidney failure; Etc.	I12-I13; N00-N05; N07; N11; N14; N17-N19; Q61

Appendix Table B – International Classification of Disease 10th Edition (ICD-10) codes used for defining any hospital-treated infection

Infection Type	ICD10	Exclusion
Abscess	A541	A541B
Abscess	B43	B430, B438, B439
Abscess	D733	
Abscess	E060A	
Abscess	E236A	
Abscess	E321	
Abscess	H000A	
Abscess	H050A	
Abscess	H440A	
Abscess	H600	
Abscess	H600A	
Abscess	J340	J340E, J340F, J340G, J340H
Abscess	J340A	
Abscess	J36	
Abscess	J383D	
Abscess	J387G	
Abscess	J390	
Abscess	J391	
Abscess	J398A	
Abscess	J851	
Abscess	J852	
Abscess	J853	
Abscess	K046	
Abscess	K047	
Abscess	K113	
Abscess	K122	
Abscess	K130A	
Abscess	K140A	
Abscess	K209A	
Abscess	K353A	
Abscess	K353B	
Abscess	K570	K570B, K570C
Abscess	K572	K572B, K572C
Abscess	K574	K574A
Abscess	K578	
Abscess	K61	
Abscess	K630	
Abscess	K750	
Abscess	K810A	
Abscess	K858A	
Abscess	L02	
Abscess	L050	
Abscess	M608A	
Abscess	M608A1	
Abscess	M868A	
Abscess	M869A	
Abscess	N151	
Abscess	N340	
Abscess	N412	
Abscess	N450	
Abscess	N492A	
Abscess	N619A	
Abscess	N619B	
Abscess	N700A	
Abscess	N700B	

Abscess	N710A
Abscess	N730A
Abscess	N730B
Abscess	N732A
Abscess	N732B
Abscess	N733A
Abscess	N735A
Abscess	N738A
Abscess	N738C
Abscess	N751
Abscess	N764
Abscess	N768A
Bacteremia	A394
Bacteremia	A499A
Candidiasis and other fungal infections	B35
Candidiasis and other fungal infections	B36
Candidiasis and other fungal infections	B37
Candidiasis and other fungal infections	B38
Candidiasis and other fungal infections	B39
Candidiasis and other fungal infections	B40
Candidiasis and other fungal infections	B41
Candidiasis and other fungal infections	B42
Candidiasis and other fungal infections	B43
Candidiasis and other fungal infections	B44
Candidiasis and other fungal infections	B45
Candidiasis and other fungal infections	B46
Candidiasis and other fungal infections	B47
Candidiasis and other fungal infections	B48
Candidiasis and other fungal infections	B49
COVID-19	B342A
COVID-19	B948A
COVID-19	B972A
COVID-19	Z861A
Female pelvic infections	N70
Female pelvic infections	N71
Female pelvic infections	N72
Female pelvic infections	N73
Female pelvic infections	N74
Female pelvic infections	N75
Female pelvic infections	N76
Female pelvic infections	N77
Heart infections (acute rheumatic fever, infectious peri- or myocarditis, endocarditis)	I00
Heart infections (acute rheumatic fever, infectious peri- or myocarditis, endocarditis)	I01
Heart infections (acute rheumatic fever, infectious peri- or myocarditis, endocarditis)	I02
Heart infections (acute rheumatic fever, infectious peri- or myocarditis, endocarditis)	I301
Heart infections (acute rheumatic fever, infectious peri- or myocarditis, endocarditis)	I320
Heart infections (acute rheumatic fever, infectious peri- or myocarditis, endocarditis)	I33
Heart infections (acute rheumatic fever, infectious peri- or myocarditis, endocarditis)	I38
Heart infections (acute rheumatic fever, infectious peri- or myocarditis, endocarditis)	I398
Heart infections (acute rheumatic fever, infectious peri- or myocarditis, endocarditis)	I400
Human immunodeficiency virus [HIV] disease	B20
Human immunodeficiency virus [HIV] disease	B21

Human immunodeficiency virus [HIV] disease	B22
Human immunodeficiency virus [HIV] disease	B23
Human immunodeficiency virus [HIV] disease	B24
Infections of CNS	A022C
Infections of CNS	A170
Infections of CNS	A203
Infections of CNS	A321
Infections of CNS	A390
Infections of CNS	A548D
Infections of CNS	A80
Infections of CNS	A81
Infections of CNS	A82
Infections of CNS	A83
Infections of CNS	A84
Infections of CNS	A85
Infections of CNS	A86
Infections of CNS	A87
Infections of CNS	A88
Infections of CNS	A89
Infections of CNS	G00
Infections of CNS	G01
Infections of CNS	G02
Infections of CNS	G03
Infections of CNS	G04
Infections of CNS	G05
Infections of CNS	G06
Infections of CNS	G07
Infectious complications of procedures, catheters etc.	T802
Infectious complications of procedures, catheters etc.	T814
Infectious complications of procedures, catheters etc.	T826
Infectious complications of procedures, catheters etc.	T827
Infectious complications of procedures, catheters etc.	T835
Infectious complications of procedures, catheters etc.	T836
Infectious complications of procedures, catheters etc.	T845
Infectious complications of procedures, catheters etc.	T846
Infectious complications of procedures, catheters etc.	T847
Infectious complications of procedures, catheters etc.	T857
Infectious complications of procedures, catheters etc.	T880
Infectious complications of procedures, catheters etc.	T880A
Infectious complications of procedures, catheters etc.	T89
Infectious complications of procedures, catheters etc.	T899
Influenza	J10
Influenza	J11
Intra-abdominal infections	A00
Intra-abdominal infections	A01
Intra-abdominal infections	A02
Intra-abdominal infections	A03
Intra-abdominal infections	A04
Intra-abdominal infections	A05
Intra-abdominal infections	A06
Intra-abdominal infections	A07
Intra-abdominal infections	A08
Intra-abdominal infections	A09
Intra-abdominal infections	K35
Intra-abdominal infections	K37
Intra-abdominal infections	K570
Intra-abdominal infections	K572
Intra-abdominal infections	K574
Intra-abdominal infections	K578

Intra-abdominal infections	K61	
Intra-abdominal infections	K630	
Intra-abdominal infections	K650	K650M, K650N, K650O, K650P
Intra-abdominal infections	K659	
Intra-abdominal infections	K67	
Intra-abdominal infections	K750	
Intra-abdominal infections	K751	
Intra-abdominal infections	K800	
Intra-abdominal infections	K803	
Intra-abdominal infections	K804	
Intra-abdominal infections	K810	
Intra-abdominal infections	K819	
Intra-abdominal infections	K830	
Intra-abdominal infections	K858A	
Intra-abdominal infections	K859	
Male genital infections	N41	
Male genital infections	N45	
Miscellaneous bacterial infections	A20	
Miscellaneous bacterial infections	A21	
Miscellaneous bacterial infections	A22	
Miscellaneous bacterial infections	A23	
Miscellaneous bacterial infections	A24	
Miscellaneous bacterial infections	A25	
Miscellaneous bacterial infections	A26	
Miscellaneous bacterial infections	A27	
Miscellaneous bacterial infections	A28	
Miscellaneous bacterial infections	A30	
Miscellaneous bacterial infections	A31	
Miscellaneous bacterial infections	A32	
Miscellaneous bacterial infections	A33	
Miscellaneous bacterial infections	A34	
Miscellaneous bacterial infections	A35	
Miscellaneous bacterial infections	A36	
Miscellaneous bacterial infections	A37	
Miscellaneous bacterial infections	A38	
Miscellaneous bacterial infections	A42	
Miscellaneous bacterial infections	A43	
Miscellaneous bacterial infections	A44	
Miscellaneous bacterial infections	A48	
Miscellaneous bacterial infections	A49	
Miscellaneous bacterial infections	A65	
Miscellaneous bacterial infections	A66	
Miscellaneous bacterial infections	A67	
Miscellaneous bacterial infections	A68	
Miscellaneous bacterial infections	A69	
Miscellaneous bacterial infections	A70	
Miscellaneous bacterial infections	A71	
Miscellaneous bacterial infections	A74	
Miscellaneous bacterial infections	A75	
Miscellaneous bacterial infections	A77	
Miscellaneous bacterial infections	A78	
Miscellaneous bacterial infections	A79	
Miscellaneous viral infections	B00	
Miscellaneous viral infections	B01	
Miscellaneous viral infections	B02	
Miscellaneous viral infections	B03	
Miscellaneous viral infections	B04	
Miscellaneous viral infections	B05	
Miscellaneous viral infections	B06	

Miscellaneous viral infections	B07	
Miscellaneous viral infections	B08	
Miscellaneous viral infections	B09	
Miscellaneous viral infections	B25	
Miscellaneous viral infections	B26	
Miscellaneous viral infections	B27	
Miscellaneous viral infections	B30	
Miscellaneous viral infections	B33	
Miscellaneous viral infections	B34	
Obstetrical infections	O23	
Obstetrical infections	O264	
Obstetrical infections	O411	
Obstetrical infections	O740	
Obstetrical infections	O753	
Obstetrical infections	O85	
Obstetrical infections	O86	
Obstetrical infections	O883	
Obstetrical infections	O91	
Obstetrical infections	O98	
Other lower-respiratory tract infections	J20	
Other lower-respiratory tract infections	J21	
Other lower-respiratory tract infections	J22	
Other lower-respiratory tract infections	J340	J340E, J340F, J340G, J340H
Other lower-respiratory tract infections	J350	
Other lower-respiratory tract infections	J383C	
Other lower-respiratory tract infections	J383D	
Other lower-respiratory tract infections	J387B	
Other lower-respiratory tract infections	J387F	
Other lower-respiratory tract infections	J387G	
Other lower-respiratory tract infections	J440	
Other lower-respiratory tract infections	J851	
Other lower-respiratory tract infections	J86	
Other or sequelae	B90	
Other or sequelae	B91	
Other or sequelae	B92	
Other or sequelae	B94	B948A
Other or sequelae	B95	
Other or sequelae	B96	
Other or sequelae	B97	B972A
Other or sequelae	B98	
Other or sequelae	B99	
Other or sequelae	H00	H000A, H010
Other or sequelae	H030	
Other or sequelae	H031	
Other or sequelae	H043	
Other or sequelae	H050	H050A
Other or sequelae	H061	
Other or sequelae	H10	
Other or sequelae	H130	
Other or sequelae	H131	
Other or sequelae	H150	
Other or sequelae	H191	
Other or sequelae	H192	
Other or sequelae	H220	
Other or sequelae	H320	
Other or sequelae	H440	H440A
Other or sequelae	H441	
Other or sequelae	H609	
Other or sequelae	H610	
Other or sequelae	H620	

Other or sequelae	H621	
Other or sequelae	H622	
Other or sequelae	H623	
Other or sequelae	H650	
Other or sequelae	H660	
Other or sequelae	H661	
Other or sequelae	H662	
Other or sequelae	H663	
Other or sequelae	H664	
Other or sequelae	H669	
Other or sequelae	H67	
Other or sequelae	H680	
Other or sequelae	H700	
Other or sequelae	H830	
Other or sequelae	H940	
Other or sequelae	K040	
Other or sequelae	K052	
Other or sequelae	L04	
Other or sequelae	L05	L050
Other or sequelae	N481	
Other or sequelae	N482	
Other or sequelae	N49	N492A
Other or sequelae	N510	
Other or sequelae	N511	
Other or sequelae	N512	
Other or sequelae	N619E	
Parasitic infections	B50	
Parasitic infections	B51	
Parasitic infections	B52	
Parasitic infections	B53	
Parasitic infections	B54	
Parasitic infections	B55	
Parasitic infections	B56	
Parasitic infections	B57	
Parasitic infections	B58	
Parasitic infections	B60	
Parasitic infections	B64	
Parasitic infections	B65	
Parasitic infections	B66	
Parasitic infections	B67	
Parasitic infections	B68	
Parasitic infections	B69	
Parasitic infections	B70	
Parasitic infections	B71	
Parasitic infections	B72	
Parasitic infections	B73	
Parasitic infections	B74	
Parasitic infections	B75	
Parasitic infections	B76	
Parasitic infections	B77	
Parasitic infections	B78	
Parasitic infections	B79	
Parasitic infections	B80	
Parasitic infections	B81	
Parasitic infections	B82	
Parasitic infections	B83	
Parasitic infections	B85	
Parasitic infections	B86	
Parasitic infections	B87	
Parasitic infections	B88	

Parasitic infections	B89	
Pneumonia	J12	
Pneumonia	J13	
Pneumonia	J14	
Pneumonia	J15	
Pneumonia	J16	
Pneumonia	J17	
Pneumonia	J18	
Sepsis	A021	
Sepsis	A227	
Sepsis	A282B	
Sepsis	A327	
Sepsis	A40	
Sepsis	A41	
Sepsis	A427	
Sepsis	A548G	
Sexually transmitted diseases	A50	
Sexually transmitted diseases	A51	
Sexually transmitted diseases	A52	
Sexually transmitted diseases	A53	
Sexually transmitted diseases	A54	
Sexually transmitted diseases	A55	
Sexually transmitted diseases	A56	
Sexually transmitted diseases	A57	
Sexually transmitted diseases	A58	
Sexually transmitted diseases	A59	
Sexually transmitted diseases	A60	
Sexually transmitted diseases	A63	
Sexually transmitted diseases	A64	
Skin infections	A46	
Skin infections	H010	
Skin infections	H03	H030, H031
Skin infections	H601	
Skin infections	H603	
Skin infections	K122	
Skin infections	K130A	
Skin infections	K61	
Skin infections	L00	
Skin infections	L01	
Skin infections	L02	
Skin infections	L03	
Skin infections	L08	
Skin infections	L303	
Skin infections	L738	
Skin infections	M726	
Spectic arthritis, osteomyelitis, myositis	M00	
Spectic arthritis, osteomyelitis, myositis	M01	
Spectic arthritis, osteomyelitis, myositis	M630	
Spectic arthritis, osteomyelitis, myositis	M631	
Spectic arthritis, osteomyelitis, myositis	M632	
Spectic arthritis, osteomyelitis, myositis	M86	
Upper respiratory tract infection	J00	
Upper respiratory tract infection	J01	
Upper respiratory tract infection	J02	
Upper respiratory tract infection	J03	
Upper respiratory tract infection	J04	
Upper respiratory tract infection	J05	
Upper respiratory tract infection	J06	
Upper respiratory tract infection	J36	
Upper respiratory tract infection	J390	

Upper respiratory tract infection	J391
Urinary tract infections	N080
Urinary tract infections	N10
Urinary tract infections	N11
Urinary tract infections	N12
Urinary tract infections	N136
Urinary tract infections	N151
Urinary tract infections	N159
Urinary tract infections	N160
Urinary tract infections	N288D
Urinary tract infections	N288E
Urinary tract infections	N288F
Urinary tract infections	N290
Urinary tract infections	N291
Urinary tract infections	N30
Urinary tract infections	N330
Urinary tract infections	N340
Urinary tract infections	N341
Urinary tract infections	N390
Viral hepatitis	B15
Viral hepatitis	B16
Viral hepatitis	B17
Viral hepatitis	B18
Viral hepatitis	B19

Appendix Table C – International Classification of Disease 10th Edition (ICD-10) codes used for defining any hospital-treated pneumonia and hospital-treated urinary tract infection

**Outcome**	**Code**	**System**	**Text**	**Comments**
**Hospital-treated pneumonia**				
	J12	ICD-10	Viral pneumonia, not elsewhere classified	
	J13	ICD-10	Pneumonia due to streptococcus pneumoniae	
	J14	ICD-10	Pneumonia due to hemophilus influenzae	
	J15	ICD-10	Bacterial pneumonia, not elsewhere classified	
	J16	ICD-10	Pneumonia due to other infectious organism, not elsewhere classified	
	J17	ICD-10	Pneumonia in disease, not elsewhere classified	
	J18	ICD-10	Pneumonia, unspecified organism	
	J69	ICD-10	Pneumonitis due to inhalation of solids and liquids	
	J85.1	ICD-10	Abscess of the lung with pneumonia	
	T81.4P	ICD-10	Pneumonia following a procedure	
**Hospital-treated urinary tract infection**				
	N080	ICD-10	Glomerular disorders in infectious and parasitic diseases classified elsewhere	
	N10	ICD-10	Acute tubule-interstitial nephritis	
	N11	ICD-10	Chronic tubulo-interstitial nephritis	
	N12	ICD-10	Tubulo-interstitial nephritis, not specified as acute or chronic	Excluding N301, N302, N304
	N13.6	ICD-10	Pyonephritis	
	N15.1	ICD-10	Renal and perinephric abscess	
	N159	ICD-10	Renal tubulo-interstitial disease, unspecified	
	N160	ICD-10	Renal tubulo-interstitial disorders in infectious and parasitic diseases classified elsewhere	
	N28.8D	ICD-10	Pyelitis cystica	
	N288E	ICD-10	Pyeloureteritis cystica	
	N288F	ICD-10	Ureteritis cystica	
	N290	ICD-10	Late syphilis of kidney	
	N291	ICD-10	Other disorders of kidney and ureter in infectious and parasitic diseases classified elsewhere	
	N30	ICD-10	Cystitis	
	N33.0	ICD-10	Tuberculous cystitis	
	N34.0	ICD-10	Urethal abscess	
	N341	ICD-10	Urethritis and urethral syndrome	
	N39.0	ICD-10	Urinary tract infection, site not specified	
	T81.4U	ICD-10	Urinary tract infection, following a procedure	
