# Supplementary material for: Major comorbid diseases as predictors of infection in the first month after hip fracture surgery: a population-based cohort study in 92,239 patients
Source: Eur Geriatr Med. 2024 May 22;15(4):1069–80. doi: 10.1007/s41999-024-00989-w (PMC11377556; doi:10.1007/s41999-024-00989-w)
Supplement: Supplementary file 2 — Supplementary file2 (PDF 235 KB) [file 41999_2024_989_MOESM2_ESM.pdf]

Online Supplementary Material 2

Journal

European Geriatric Medicine

Article title

Major comorbid diseases as predictors of infection in the first month after hip fracture surgery: a population-based cohort study in 92,239 patients

Authors

Nadia Roldsgaard Gadgaard[1], Claus Varnum[2,3], Rob Nelissen[4], Christina Vandenbroucke-Grauls[1,5], Henrik Toft Sørensen[1], and Alma B. Pedersen[1]

[1] Department of Clinical Epidemiology, Aarhus University Hospital and Aarhus University, Denmark

[2] Department of Orthopedic Surgery, Lillebaelt Hospital – Vejle, Denmark

[3] Department of Regional Health Research, University of Southern Denmark, Denmark

[4] Department of Orthopedics, Leiden University Medical Center, The Netherlands

[5] Department of Medical Microbiology and Infection Control, Amsterdam University Medical Center, Amsterdam, The Netherlands

Corresponding Authors

Nadia Roldsgaard Gadgaard, MD, PhD student

Department of Clinical Epidemiology

Aarhus University and Aarhus University Hospital

Olof Palmes Allé 43-45

8200 Aarhus N, Denmark

E-mail: nrg@clin.au.dk

Supplementary Table S1. 30-day cumulative incidence of pneumonia (total and stratified by no comorbid disease or selected comorbid diseases). Crude and adjusted odds ratios for pneumonia, comparing patients with vs. without a selected comorbid disease, are shown.

	Number at risk	Number with pneumonia	Cumulative incidence (95% CI)	Crude OR (95% CI)	Adjusted OR (95% CI)*
Total population	92,239	5,784	6.3 (6.1–6.4)	-	-
No comorbid disease	33,341	1,443	4.3 (4.1–4.6)	-	-
**Comorbid diseases**					
Cardiovascular:					
Cerebrovascular disease	12,796	977	7.6 (7.2–8.1)	1.28 (1.19–1.38)	1.04 (0.96–1.12)
Heart arrhythmia	13,967	1,194	8.5 (8.1–9.0)	1.50 (1.40–1.60)	1.10 (1.02–1.18)
Heart failure	6,850	640	9.3 (8.7–10.0)	1.61 (1.47–1.75)	1.04 (0.95–1.15)
Hypertension	21,235	1,610	7.6 (7.2–7.9)	1.31 (1.24–1.39)	1.08 (1.01–1.16)
Hypotension	1,427	147	10.3 (8.8–11.9)	1.74 (1.45–2.06)	1.20 (1.00–1.43)
Myocardial infarction	3,193	257	8.0 (7.1–9.0)	1.32 (1.16–1.50)	0.98 (0.85–1.12)
Peripheral vascular disease	5,476	479	8.7 (8.0–9.5)	1.47 (1.33–1.62)	1.16 (1.05–1.29)
Valvular heart disease	3,877	339	8.7 (7.9–9.7)	1.46 (1.30–1.63)	1.15 (1.02–1.29)
Hepatic/gastrointestinal					
Liver disease	1,045	60	5.7 (4.4–7.3)	0.91 (0.69–1.17)	0.83 (0.62–1.09)
Peptic ulcer	3,276	250	7.6 (6.8–8.6)	1.25 (1.09–1.42)	1.01 (0.88–1.15)
Malignant:					
Any solid tumor	9,297	610	6.6 (6.1–7.1)	1.06 (0.97–1.15)	0.90 (0.82–0.98)
Hematologic cancer	1,080	84	7.8 (6.3–9.5)	1.26 (1.00–1.57)	1.18 (0.93–1.48)
Metastatic solid tumor	1,209	77	6.4 (5.1–7.8)	1.02 (0.80–1.27)	1.03 (0.80–1.29)
Metabolic:					
Diabetes, complicated	3,621	313	8.6 (7.8–9.6)	1.44 (1.27–1.62)	1.15 (1.02–1.31)
Diabetes, uncomplicated	3,927	264	6.7 (6.0–7.5)	1.08 (0.95–1.23)	0.98 (0.86–1.12)
Hypercholesterolemia	4,420	300	6.8 (6.1–7.6)	1.09 (0.97–1.23)	0.87 (0.76–0.99)
Thyroid disease	3,492	247	7.1 (6.3–8.0)	1.14 (1.00–1.30)	1.09 (0.95–1.25)
Musculoskeletal:					
Rheumatic disease	3,410	233	6.8 (6.0–7.7)	1.10 (0.96–1.26)	1.12 (0.97–1.29)
Neurological/psychological:					
Alcohol use disorder	2,245	174	7.8 (6.7–8.9)	1.26 (1.08–1.47)	1.12 (0.94–1.33)
Dementia	8,074	616	7.6 (7.1–8.2)	1.26 (1.16–1.38)	1.09 (1.00–1.19)
Depression/anxiety	4,151	383	9.2 (8.4–10.1)	1.56 (1.39–1.73)	1.30 (1.16–1.46)
Neurological disorder	4,371	358	8.2 (7.4–9.0)	1.36 (1.21–1.51)	1.29 (1.14–1.44)
Pulmonary:					
Chronic pulmonary disease	9,647	1,189	12.3 (11.7–13.0)	2.39 (2.23–2.55)	2.22 (2.07–2.39)
Pulmonary circulation disorder	1,388	137	9.9 (8.4–11.5)	1.65 (1.38–1.97)	1.20 (0.99–1.43)
Renal/hematological:					
Anemia	8,364	710	8.5 (7.9–9.1)	1.44 (1.33–1.56)	1.09 (1.00–1.19)
Fluid and electrolyte disorder	9,700	869	9.0 (8.4–9.5)	1.55 (1.44–1.67)	1.23 (1.13–1.33)
Renal disease	3,159	331	10.5 (9.4–11.6)	1.79 (1.59–2.01)	1.16 (1.03–1.32)

*Adjusted by age, sex, and comorbid diseases.
Abbreviations: odds ratio (OR); confidence interval (CI).

Supplementary Table S2. 30-day cumulative incidence of urinary tract infection (total and stratified by no comorbid disease and selected comorbid diseases). Crude and adjusted odds ratios for urinary tract infection, comparing patients with vs. without a selected comorbid disease, are shown.

	Number at risk	Number with urinary tract infection	Cumulative incidence (95% CI)	Crude OR (95% CI)	Adjusted OR (95% CI)*
Total population	92,239	6,614	7.2 (7.0–7.3)	-	-
No comorbid disease	33,341	2,105	6.3 (6.1–6.6)	-	-
**Comorbid diseases**					
Cardiovascular:					
Cerebrovascular disease	12,796	1,079	8.4 (8.0–8.9)	1.23 (1.15–1.32)	1.13 (1.05–1.22)
Heart arrhythmia	13,967	1,152	8.2 (7.8–8.7)	1.20 (1.12–1.28)	1.06 (0.99–1.14)
Heart failure	6,850	539	7.9 (7.2–8.5)	1.12 (1.02–1.22)	0.96 (0.86–1.06)
Hypertension	21,235	1,767	8.3 (8.0–8.7)	1.24 (1.17–1.31)	1.06 (0.99–1.13)
Hypotension	1,427	143	10.0 (8.5–11.6)	1.45 (1.21–1.72)	1.18 (0.99–1.41)
Myocardial infarction	3,193	253	7.9 (7.0–8.9)	1.12 (0.98–1.27)	1.02 (0.89–1.16)
Peripheral vascular disease	5,476	397	7.2 (6.6–8.0)	1.01 (0.91–1.12)	1.00 (0.89–1.11)
Valvular heart disease	3,877	288	7.4 (6.6–8.3)	1.04 (0.92–1.17)	0.90 (0.79–1.02)
Hepatic/gastrointestinal					
Liver disease	1,045	87	8.3 (6.8–10.1)	1.18 (0.94–1.46)	1.27 (1.00–1.60)
Peptic ulcer	3,276	270	8.2 (7.3–9.2)	1.17 (1.03–1.33)	1.08 (0.94–1.22)
Malignant:					
Any solid tumor	9,297	642	6.9 (6.4–7.4)	0.96 (0.88–1.04)	0.96 (0.88–1.04)
Hematologic cancer	1,080	79	7.3 (5.9–9.0)	1.02 (0.81–1.28)	1.07 (0.84–1.34)
Metastatic solid tumor	1,209	64	5.3 (4.1–6.7)	0.72 (0.55–0.92)	0.81 (0.62–1.04)
Metabolic:					
Diabetes, complicated	3,621	327	9.0 (8.1–10.0)	1.30 (1.16–1.46)	1.33 (1.18–1.51)
Diabetes, uncomplicated	3,927	340	8.7 (7.8–9.6)	1.24 (1.10–1.39)	1.23 (1.10–1.38)
Hypercholesterolemia	4,420	374	8.5 (7.7–9.3)	1.21 (1.08–1.35)	1.09 (0.97–1.23)
Thyroid disease	3,492	318	9.1 (8.2–10.1)	1.31 (1.16–1.47)	1.09 (0.97–1.23)
Musculoskeletal:					
Rheumatic disease	3,410	259	7.6 (6.7–8.5)	1.07 (0.94–1.21)	1.02 (0.89–1.16)
Neurological/psychological:					
Alcohol use disorder	2,245	150	6.7 (5.7–7.8)	0.93 (0.78–1.09)	1.08 (0.90–1.29)
Dementia	8,074	645	8.0 (7.4–8.6)	1.14 (1.04–1.24)	0.96 (0.88–1.05)
Depression/anxiety	4,151	447	10.8 (9.8–11.7)	1.60 (1.45–1.77)	1.41 (1.26–1.56)
Neurological disorder	4,371	378	8.6 (7.8–9.5)	1.24 (1.11–1.38)	1.32 (1.18–1.48)
Pulmonary:					
Chronic pulmonary disease	9,647	734	7.6 (7.1–8.1)	1.07 (0.99–1.16)	1.09 (1.00–1.18)
Pulmonary circulation disorder	1,388	112	8.1 (6.7–9.6)	1.14 (0.93–1.38)	1.03 (0.84–1.26)
Renal/hematological:					
Anemia	8,364	739	8.8 (8.2–9.5)	1.29 (1.19–1.39)	1.12 (1.03–1.22)
Fluid and electrolyte disorder	9,700	881	9.1 (8.5–9.7)	1.34 (1.24–1.44)	1.09 (1.01–1.18)
Renal disease	3,159	282	8.9 (8.0–10.0)	1.28 (1.13–1.45)	1.18 (1.04–1.35)

*Adjusted by age, sex, and comorbid diseases.
Abbreviations: odds ratio (OR); confidence interval (CI).

Supplementary Table S3. 30-day cumulative incidence with 95% confidence intervals for any infection (total and stratified by no comorbid disease or selected comorbid diseases, age group and sex).

	Age 65–74	Age 75–84	Age ≥85	Male	Female
Total	11.8 (11.4–12.3)	15.1 (14.7–15.5)	17.3 (16.9–17.7)	17.7 (17.2–18.1)	14.5 (14.2–14.7)
No comorbid disease	7.8 (7.2–8.5)	11.2 (10.7–11.8)	15.1 (14.5–15.7)	13.9 (13.2–14.7)	11.6 (11.2–12.0)
**Comorbid diseases**					
Cardiovascular:					
Cerebrovascular disease	15.7 (14.3–17.1)	17.9 (16.9–18.9)	19.2 (18.1–20.3)	20.3 (19.2–21.4)	16.6 (15.8–17.4)
Heart arrhythmia	17.1 (15.3–18.9)	19.3 (18.3–20.4)	19.5 (18.6–20.4)	21.5 (20.4–22.7)	17.8 (17.1–18.6)
Heart failure	18.5 (16.1–21.0)	21.1 (19.5–22.7)	19.8 (18.4–21.1)	21.4 (19.8–22.9)	19.3 (18.1–20.5)
Hypertension	15.6 (14.5–16.8)	18.2 (17.4–19.0)	19.9 (19.1–20.7)	20.9 (19.9–21.9)	17.5 (16.9–18.1)
Hypotension	17.5 (12.7–23.0)	22.3 (18.9–25.8)	23.6 (20.5–26.9)	23.6 (20.1–27.3)	21.3 (18.7–24.1)
Myocardial infarction	18.0 (14.8–21.5)	19.3 (17.1–21.5)	19.6 (17.5–21.7)	20.5 (18.3–22.8)	18.4 (16.7–20.2)
Peripheral vascular disease	14.9 (13.0–17.0)	19.1 (17.5–20.6)	19.7 (17.9–21.7)	20.8 (19.2–22.5)	16.5 (15.3–17.8)
Valvular heart disease	16.2 (12.8–19.8)	18.7 (16.8–20.7)	19.6 (17.8–21.4)	21.5 (19.2–23.8)	17.7 (16.3–19.2)
Hepatic/gastrointestinal					
Liver disease	16.6 (13.6–19.9)	17.8 (14.0–22.0)	20.8 (14.6–27.8)	18.6 (15.0–22.6)	17.0 (14.2–20.0)
Peptic ulcer	17.2 (14.4–20.2)	19.8 (17.7–22.0)	17.1 (15.1–19.2)	21.0 (18.7–23.5)	16.8 (15.2–18.4)
Malignant:					
Any solid tumor	12.7 (11.3–14.3)	15.7 (14.6–16.9)	16.9 (15.7–18.2)	17.2 (16.0–18.5)	14.6 (13.7–15.5)
Hematologic cancer	18.6 (14.0–23.6)	17.4 (14.2–20.9)	20.0 (16.0–24.4)	19.2 (15.4–23.2)	18.1 (15.4–21.1)
Metastatic solid tumor	12.3 (9.5–15.4)	15.1 (12.2–18.4)	18.4 (13.7–23.7)	15.1 (11.9–18.5)	14.4 (12.0–17.0)
Metabolic:					
Diabetes, complicated	19.6 (17.3–22.1)	21.6 (19.6–23.7)	21.1 (18.6–23.8)	22.6 (20.6–24.7)	19.5 (17.8–21.3)
Diabetes, uncomplicated	14.3 (12.0–16.6)	18.1 (16.3–20.0)	19.6 (17.5–21.8)	19.8 (17.7–22.0)	16.8 (15.4–18.2)
Hypercholesterolemia	15.2 (13.2–17.4)	17.6 (16.0–19.3)	20.6 (18.4–22.8)	19.7 (17.8–21.7)	16.9 (15.5–18.3)
Thyroid disease	17.8 (14.7–21.2)	16.3 (14.4–18.3)	19.5 (17.5–21.5)	19.6 (16.0–23.6)	17.7 (16.4–19.1)
Musculoskeletal:					
Rheumatic disease	13.8 (11.4–16.3)	17.1 (15.3–19.0)	19.9 (17.6–22.3)	18.3 (15.4–21.3)	17.0 (15.6–18.4)
Neurological/psychological:					
Alcohol use disorder	16.4 (14.5–18.3)	19.2 (16.3–22.3)	24.0 (17.8–30.7)	18.2 (16.2–20.4)	17.2 (14.9–19.6)
Dementia	19.9 (17.0–23.0)	18.1 (16.8–19.5)	16.8 (15.7–17.9)	21.5 (19.8–23.2)	16.0 (15.1–17.0.)
Depression/anxiety	21.0 (18.1–24.0)	23.4 (21.5–25.4)	22.5 (20.5–24.5)	26.1 (23.4–28.8)	21.5 (20.1–22.9)
Neurological disorder	15.6 (13.7–17.6)	19.5 (17.8–21.3)	20.7 (18.3–23.2)	20.0 (18.2–21.9)	17.6 (16.1–19.1)
Pulmonary:					
Chronic pulmonary disease	19.7 (18.1–21.4)	22.9 (21.7–24.1)	24.3 (22.8–25.9)	25.4 (23.9–26.9)	21.0 (20.0–22.0)
Pulmonary circulation disorder	17.4 (13.1–22.3)	18.4 (15.4–21.7)	22.4 (19.1–26.0)	23.8 (19.8–27.9)	18.2 (15.8–20.7)
Renal/hematological:					
Anemia	18.9 (16.9–21.0)	20.9 (19.5–22.4)	19.3 (18.1–20.5)	21.8 (20.3–23.3)	18.9 (17.9–19.9)
Fluid and electrolyte disorder	18.7 (16.6–20.8)	21.5 (20.1–22.9)	20.2 (19.1–21.3)	23.6 (22.0–25.2)	19.2 (18.3–20.2)
Renal disease	22.9 (19.7–26.4)	25.0 (22.7–27.3)	23.3 (20.9–25.7)	25.9 (23.8–28.1)	21.9 (19.9–24.0)

Supplementary Table S4. Adjusted odds ratios with 95% confidence intervals for any infection, comparing patients with vs. without a selected comorbid disease, mutually adjusted by age, sex, and comorbid diseases, and stratified by age group and sex.

	Age 65–74	Age 75–84	Age ≥85	Male	Female
**Comorbid diseases**					
Cardiovascular:					
Cerebrovascular disease	1.13 (0.99–1.28)	1.05 (0.96–1.14)	1.02 (0.94–1.11)	1.10 (1.01–1.19)	1.02 (0.95–1.09)
Heart arrhythmia	1.15 (0.98–1.33)	1.13 (1.04–1.23)	1.06 (0.98–1.13)	1.12 (1.03–1.22)	1.07 (1.01–1.15)
Heart failure	1.01 (0.82–1.23)	1.10 (0.98–1.22)	0.99 (0.89–1.09)	0.97 (0.87–1.08)	1.07 (0.98–1.17)
Hypertension	1.09 (0.96–1.23)	1.08 (1.01–1.17)	1.15 (1.07–1.23)	1.08 (1.00–1.18)	1.13 (1.07–1.19)
Hypotension	0.94 (0.64–1.35)	1.13 (0.92–1.39)	1.24 (1.03–1.49)	1.12 (0.90–1.37)	1.20 (1.01–1.41)
Myocardial infarction	1.16 (0.89–1.48)	1.03 (0.88–1.19)	1.03 (0.90–1.18)	1.00 (0.86–1.16)	1.09 (0.96–1.23)
Peripheral vascular disease	0.98 (0.82–1.16)	1.09 (0.97–1.21)	1.04 (0.92–1.18)	1.09 (0.97–1.22)	1.04 (0.94–1.15)
Valvular heart disease	0.97 (0.73–1.28)	1.04 (0.90–1.19)	1.04 (0.92–1.17)	1.05 (0.91–1.22)	1.03 (0.93–1.15)
Hepatic/gastrointestinal					
Liver disease	1.13 (0.87–1.46)	1.04 (0.78–1.38)	1.11 (0.72–1.64)	1.07 (0.81–1.41)	1.12 (0.90–1.40)
Peptic ulcer	1.12 (0.90–1.39)	1.14 (0.99–1.32)	0.90 (0.77–1.04)	1.08 (0.93–1.26)	1.01 (0.90–1.14)
Malignant:					
Any solid tumor	0.99 (0.85–1.14)	0.95 (0.86–1.04)	0.90 (0.82–0.98)	0.90 (0.82–0.99)	0.96 (0.89–1.04)
Hematologic cancer	1.59 (1.13–2.19)	1.11 (0.87–1.40)	1.14 (0.87–1.49)	1.10 (0.85–1.42)	1.31 (1.07–1.59)
Metastatic solid tumor	0.96 (0.72–1.27)	0.95 (0.74–1.20)	1.03 (0.72–1.42)	0.87 (0.66–1.12)	1.04 (0.84–1.27)
Metabolic:					
Diabetes, complicated	1.45 (1.22–1.72)	1.28 (1.12–1.46)	1.09 (0.93–1.29)	1.26 (1.10–1.43)	1.30 (1.15–1.46)
Diabetes, uncomplicated	1.12 (0.91–1.36)	1.14 (0.99–1.29)	1.07 (0.93–1.23)	1.13 (0.97–1.30)	1.10 (0.99–1.23)
Hypercholesterolemia	0.95 (0.79–1.15)	0.94 (0.82–1.06)	1.05 (0.91–1.22)	0.97 (0.85–1.12)	1.00 (0.90–1.11)
Thyroid disease	1.36 (1.07–1.72)	0.94 (0.81–1.09)	1.07 (0.94–1.22)	0.92 (0.72–1.18)	1.08 (0.97–1.18)
Musculoskeletal:					
Rheumatic disease	1.09 (0.87–1.34)	1.10 (0.95–1.25)	1.14 (0.98–1.32)	0.96 (0.78–1.17)	1.16 (1.05–1.29)
Neurological/psychological:					
Alcohol use disorder	1.12 (0.94–1.33)	1.06 (0.86–1.30)	1.27 (0.88–1.81)	1.07 (0.91–1.26)	1.24 (1.03–1.47)
Dementia	1.59 (1.29–1.94)	1.12 (1.01–1.23)	0.89 (0.81–0.97)	1.13 (1.01–1.26)	0.97 (0.90–1.05)
Depression/anxiety	1.53 (1.26–1.85)	1.45 (1.28–1.63)	1.28 (1.13–1.44)	1.40 (1.20–1.62)	1.39 (1.27–1.52)
Neurological disorder	1.24 (1.05–1.45)	1.29 (1.14–1.45)	1.16 (0.99–1.35)	1.20 (1.06–1.36)	1.30 (1.16–1.44)
Pulmonary:					
Chronic pulmonary disease	1.80 (1.60–2.03)	1.62 (1.49–1.75)	1.45 (1.32–1.60)	1.60 (1.47–1.75)	1.61 (1.50–1.72)
Pulmonary circulation disorder	1.09 (0.77–1.51)	0.95 (0.76–1.18)	1.19 (0.96–1.45)	1.22 (0.96–1.53)	1.01 (0.85–1.19)
Renal/hematological:					
Anemia	1.25 (1.07–1.46)	1.18 (1.07–1.30)	1.03 (0.94–1.12)	1.07 (0.96–1.18)	1.15 (1.06–1.23)
Fluid and electrolyte disorder	1.23 (1.04–1.43)	1.25 (1.14–1.37)	1.13 (1.04–1.22)	1.20 (1.08–1.32)	1.16 (1.08–1.24)
Renal disease	1.52 (1.22–1.87)	1.38 (1.20–1.58)	1.18 (1.02–1.35)	1.37 (1.20–1.55)	1.30 (1.14–1.47)
